# Factors Affecting Adherence, Intake, and Perceived Palatability of Oral Nutritional Supplements: A Literature Review

**DOI:** 10.1007/s12603-022-1819-3

**Published:** 2022-06-27

**Authors:** S. Lester, M. Kleijn, L. Cornacchia, L. Hewson, M.A. Taylor, Ian Fisk

**Affiliations:** 1 Division of Food Nutrition and Dietetics, School of Biosciences, University of Nottingham, Nottingham, Nottinghamshire, UK; 2 National Institute for Health Research (NIHR), Nottingham Biomedical Research Centre, Division of Physiology, Pharmacology and Neuroscience, School of Life Sciences, University of Nottingham, Nottingham, UK; 3 Danone Nutricia Research, Uppsalalaan 12, 3584 CT, Utrecht, the Netherlands; 4 The University of Adelaide, North Terrace, Adelaide, South Australia, Australia

**Keywords:** Healthy ageing, oral nutritional supplements, adherence, palatability

## Abstract

Oral nutritional supplements (ONS) are a clinically effective and relatively inexpensive way to supplement the diet of patients with, or at risk of, undernutrition. Good adherence is a primary determinant of the effectiveness of ONS. However adherence can be problematic for those with the greatest clinical need, such as undernourished older adults. This review aimed to appraise the available literature for the factors (contextual, personal and product related) affecting patient adherence and perceived palatability of ONS, identify areas requiring improvement and uncover gaps in the evidence to guide the focus of future research. Contextual factors identified were healthcare staff and the timing of administration. Personal factors included sensory changes and motivation which alter experience of and desire to consume ONS. The product's sensory characteristics determined palatability and intake, but undesirable attributes, such as off-flavours, can stem from nutritional ingredients. The contribution made by aroma to older adults' experience of ONS was a comparatively under-researched area. Further research should address this evidence gap to optimise the flavour, aroma profile and palatability for undernourished older consumers, thereby optimising intake. A combined multidisciplinary effort involving strategic expansion of research, industry development and clinical practice should simultaneously address the factors identified, to provide the best approach to improve adherence.

## Introduction

**M**alnutrition, often causing or resulting from disease, is prevalent worldwide and produces adverse functional effects with clinical and public-health consequences, including considerable associated economic demands (1). In particular, undernutrition has been identified as a prevalent problem in the growing population of older adults (2) (often defined as 65 years and older) due to factors such as appetite changes, swallowing difficulties and sensory changes. These factors can be encompassed under the common term “anorexia of ageing” (2). Associations exist between “anorexia of ageing” and decline in cognitive function, micronutrient deficiency, decrease in bone mass, weight loss, frailty and sarcopenia. The latter syndrome is demonstrated to have major detrimental impacts on quality of life (3) due to its systemic physiological impact on muscle strength and physical performance (4). The anorexia of ageing and sarcopenia are also identified as a major cause of physical frailty (5) defined as a condition in which a functional older person is at increased risk of adverse outcomes such as the onset of disability, morbidity, institutionalisation or mortality or who experience a failure to integrate adequate responses in the face of stress (6)..

Oral nutritional supplements (ONS) are a clinically effectve and relatively inexpensive way to supplement the diet of those older individuals who are undernourished or at risk of undernutrition (7). ONS are often nutritionally complete, meaning that when consumed in adequate quantities they can provide all essential nutrients (macronutrients along with essential micronutrients) to be a sole source of nutrition, which may not be achievable through a regular diet. Stratton et al (8) conducted a ‘review of reviews’ and found that ONS consistently increased total nutritional intake along with a significant overall reduction in mortality and reduction in complications (e.g. infections, pressure ulcers) across patient groups. One UK study estimated that the appropriate provision of ONS could result in net cost savings of £172–£229 million per annum, mainly due to reduced healthcare costs (9).

Adherence is a primary determinant of the effectiveness of a clinical nutritional intervention or treatment (10) and is defined as “the extent to which a person's behaviour — taking medication, following a diet, and/or executing lifestyle changes, corresponds with agreed recommendations from a health care provider”. Literature shows variations in levels of adherence to ONS, ranging from lower than desirable (11, 12, 13) to good (14). Optimising adherence to the prescribed level of intake will increase the effectiveness.

A greater understanding of the factors contributing to ONS intake in older adults should enable the development of appropriate strategies to support the health of older persons (15). This review aimed to appraise the available literature for the factors (contextual, personal and product related) affecting patient adherence and perceived palatability of ONS, identify areas requiring improvement and uncover gaps in the evidence to guide the focus of future research.

## Search Strategy

To identify studies relevant to the research aims for this review, the electronic databases PubMed, Google Scholar, Medline, ScienceDirect and The Cochrane Library were searched using a representative but non-exclusive list of search terms including “oral nutritional supplement”, “sip feed”, “adherence”, “appetite”, “older age”, “protein”, both singularly and in combination. The search output was then reviewed by the primary author and papers screened and selected based on their title and abstract having relevance to the topic. The bibliographies of selected papers, and articles that had cited key articles, were screened for further papers that were relevant. All study types (observational, randomised controlled trials, reviews, conference papers), ONS type (nutritionally complete or incomplete) and disease state (malnourished vs healthy) were included for this review. Exclusion criteria included articles not written in the English language, parenteral nutrition, studies not relating to oral nutritional supplements and/or high-protein foods or supplements and/or studies not relating to nutritional intake in the older person.

**Figure 1:**
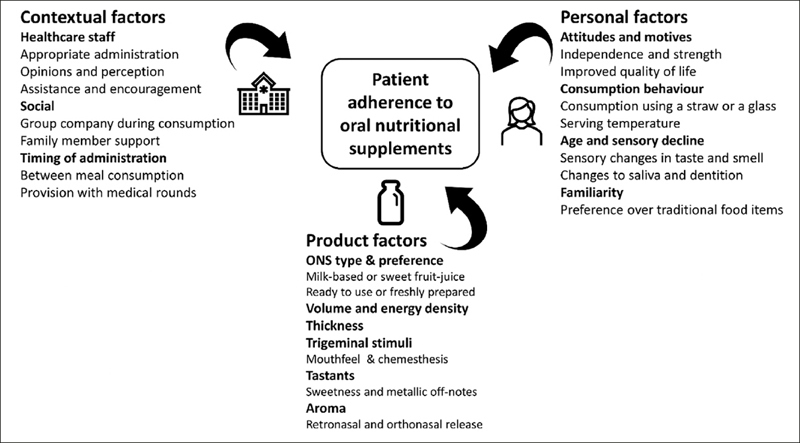
Determinants of ONS adherence, as identified in the literature

The literature review established that adherence to ONS has multifaceted determinants. However, as demonstrated in Figure 1, these can be broadly categorised into three domains: ‘contextual’ (environmental), ‘person’ and ‘product’ factors (16, 17). Categorising by these three domains, the main factors affecting ONS adherence and perceived palatability of ONS will be discussed, gaps in the literature identified and recommendations made for future research.

## Contextual factors

Contextual factors are those not directly related to the food or subject (16); they may include the physical setting of the patient, along with the interaction with those around them, such as family members and healthcare staff.

### Setting

Few studies have compared adherence to ONS by the setting. Miller et al (12) found no difference in adherence to ONS between those who spent most of the intervention period in institutional care (supervised administration via the drug cart) as opposed to the community (self-administration). Hubbard et al (14) found the mean percentage adherence to ONS in the community studies was 80.9%; significantly greater than the mean percentage adherence to ONS in hospital (67.2 %). However, no significant differences in adherence were found across settings when weighted for sample size.

### Social interaction

Due to reduced social networks and physical mobility, greater social isolation in older adults has been credited as a contributing factor to undernutrition in this population (16, 18, 19). The importance of social company during eating on nutritional intake in older adults is well documented (20, 21). For example, several researchers have found that serving food in a social dining room setting versus at the bedside of older patients is beneficial to nutritional intake and clinical outcomes such as body weight (22, 23, 24). With relevance to ONS products, McAlpine et al (25) assessed snack intake (including ONS intake) in twenty-one adults aged between 60 and 79. All participants underwent both “alone” and “group” conditions. The experimenters asked participants to invite two friends to the study in group conditions. They found that overall, energy intake was higher in the group condition for all items supporting the potential for a group condition to increase ONS intake.

The views and attitudes of family members on ONS use could be important to adherence, especially if they become the healthcare proxy for the patient. Simmons et al (26) used forced-choice questionnaires to investigate family members' preferences for nutritional interventions that aimed to improve their relatives' nutritional intake. ONS was the most frequently used nutritional intervention, but family members placed ONS second to last (out of seven nutritional interventions) in order of preference, with preference given to improving quality of food and quantity of eating support. Educating family members on the benefits of consuming adequate quantities of ONS may be a technique to encourage intake.

### Healthcare staff

Healthcare staff have a significant influence on patient's nutrient intake, but some authors report that within a medical context, nutrition can have a low priority (16, 27).

Lad et al (15) suggested that the provision of ONS is not well regulated in a number of areas including instruction, administration and provision. A qualitative study by Lambert et al (28) into patients and healthcare professionals' views on the efficacy of a specialised ONS programme in Australia, found that some staff made an individual judgment when dispensing the prescription, which led to more or less ONS being dispensed. Over two consecutive days of observation in a Californian nursing home, Simmons and Patel (29) found that fewer than 10% of patients received ONS consistent with their prescription. A longer prospective study in the United States by Kayser-Jones et al (30) found that only nine of the twenty-nine residents receiving supplements (31%) were served the correct type and number of supplements as ordered by their physicians. If healthcare staff do not provide patients with their correct prescription, adherence and clinical efficacy are restricted.

The opinions of healthcare staff on ONS could also influence patient adherence to ONS. Lad et al (15) collected self-completed questionnaire data from healthcare staff and found that a small number of staff had sampled the ONS themselves, and of these, over 50 % gave unfavourable remarks such as “horrible”, “too sickly”, and “not appetising to look at or smell”. The importance of expectation on sensory perception is well known (31), so staff perception of ONS may indirectly influence patient perception and adherence. Healthcare staff could utilise this effect for a positive benefit if staff played an active role in endorsing the product to the patient, positively conveying benefits and refraining from any negative communication, thereby enhancing their expectations before patients taste the ONS.

Nursing home residents need at least an average of 38 minutes of assistance per meal to encourage adequate food and fluid intake (32). However, one study found that healthcare staff spent less than one minute per patient encouraging consumption of ONS between meals (29). Prompting and physical assistance with feeding increases nursing and care staff time demands (33) and in reality there is uncertainty over the feasibility of adjustments with limited resources, such as healthcare funding, staffing and time.

### Timing of administration

Due to the potential impact on appetite, the timing of ONS administration, and the relationship to meal timing may affect adherence to ONS and overall nutritional intake. European Society for Clinical Nutrition and Metabolism (ESPEN) recommend that staff provide ONS to patients between meals (34). Hubbard et al (14) found that compliance was good when staff provide ONS between meals, however, when compared, the instruction to ‘take ad libitum’ resulted in greater adherence.

ONS administration with medicine rounds (such as the drug cart) could also affect adherence, because it establishes the supplement as part of medical treatment. This hypothesis is supported in randomised controlled trial by Van den Berg et al (35) who found benefits to adherence and in a study by Potter et al (36) who found a reduction in weight loss and mortality risk after providing ONS with medical rounds. However, the extent of benefits gained by administering ONS with medical rounds may depend largely on the extent of dependence on the caregiver, as discussed in the later section ‘Personal Factors’.

#### Evidence summary, evidence quality and recommendations

To summarise, healthcare staff (such as nurses) and family members play key roles in patient adherence to ONS. Active encouragement, endorsement and social support from healthcare staff, carers and family members could support optimal ONS intake. Research suggests that family members rate ONS relatively low in order of preference for nutritional interventions; benefits may be gained from educating family members, along with training of doctors and nurses, and all health care staff involved in an older persons care, on the high nutritional benefits provided from consuming ONS in line with a prescription. For those older people based in the community, carers may be of key importance as enablers of ONS consumption by ensuring it is physically accessible to the service user. Future research should address this to explore how leveraging informed attitudes and opinions of healthcare staff and family members may improve adherence to ONS. Improved medical provision, such as greater accuracy when dispensing prescriptions by medical doctors and pharmacists, will enable the patient a greater opportunity to consume nutrients at levels that are most clinically meaningful and in line with their prescription. Residential setting (i.e., hospital or community) has little impact on adherence to ONS however the timing when staff administer ONS is significant for overall intake. Promoting administration of ONS, ‘ad libitum’ or between meals, results in the largest benefits gained.

Strong evidence is in favour of the recommendation of social support and encouragement for optimal nutritional intake and strong evidence from a systematic review does support offering ONS between meals or ‘ad libitum’. Much of the remaining evidence in this section is observational in nature and collected using questionnaires, which, when compared to randomised controlled trials, is classified as lower-quality in the hierarchy of evidence. However, this research does give a first-hand account of behaviours and intake in environments such as hospitals where randomised controlled trials are not always feasible, ethical and/or may be low in ecological validity. Findings from this research therefore should inform the topic of future well-controlled and designed studies to establish firm guidelines on the best practice of ONS provision.

## Personal factors

Personal factors included patient's attitudes and motives towards consuming ONS, their consumption behaviour, health status and age.

### Attitudes and motives

Den Uijl et al (17) highlighted the lack of research into the personal factors which drive ONS consumption in older frail adults. They suggested that in some subgroups, ONS are used for the benefits of the product (such as improved health status and prolongation of independence) rather than for the product attributes (e.g. taste, volume). Using a means-end chain (MEC) method, they conducted a study to elucidate the personally relevant factors related to ONS consumption in two groups of older nutritionally frail ONS users: community-dwelling persons and care home residents. Den Uijl et al (17) rendered two hierarchical value maps (HVM), revealing that the community-dwelling group took ONS to prolong their independence and strength. In contrast, the care home group reported values related to improvements in quality of life. It would be intriguing to explore the benefit of using these consumer derived and relevant values in ONS communication strategies.

An advantage of this study was the use of in-depth interviews covering a wide range of topics, including perceived benefits of ONS. Participants were also physically exposed to their product and asked to share experiences, reducing reliance on retrospective memory. Interestingly, 80% of participants viewed ONS as a snack food rather than a medicine, something which the authors attributed to the participants preferring to be regarded as consumers rather than patients. This finding conflicts with research that found that ONS administration with medical rounds improved adherence (35, 36). This conflict may be due to the fact that Den Uijl et al (2015) included cognitively healthier older persons in their study whereas Potter et al (36) and Berg et al (35) included patients with some cognitive disorder, and therefore potentially reflects those with a greater dependence on their caregivers to receive medications and nutritional support.

### ONS consumption behaviour

Small changes in the serving of foods and drinks can affect nutritional intake. For example, simply serving food and drinks to people with Alzheimer's disease on red coloured crockery, compared with white, significantly increased the amount of food and drink consumed (37). Despite this, little research has investigated the effects of consumer behaviour on ONS adherence in older groups.

Den Ujil et al (17) found that older adults living in the community preferred to drink the ONS from the bottle using a straw because it was more convenient and more manageable. However, in care home settings, almost an equal proportion of participants drank through a straw (45 %) as directly from a glass (30 %). Variations in drinking behaviour are likely to influence adherence. A randomised controlled trial by Allen et al (38) found that undernourished older adults consumed a greater amount of ONS when it was supplied to them in their usual drinking method (glass or beaker) versus with a straw inserted directly into the container. Authors attributed this to greater familiarity, as for older people, drinking from a straw may be less familiar than drinking from a glass or beaker. However, drinking from a straw requires lip muscle strength and suction pressure. Compared to younger adults, older adults have been found to have longer suction time when drinking water using a straw (39). It is important to note that with ageing, older adults can experience systemic wasting of muscle (sarcopenia), which may impede suction ability and may be a contributing factor in preference for a glass over a straw. Nonetheless, staff should consider personal preference in drinking method when presenting ONS to encourage maximum potential consumption. With recent regulations leading to ONS manufacturers removal of single use plastic straws from bottles, it would be advisable to have sustainable alternatives available within hospital and care home settings.

Another factor that may be important in adherence to ONS is the temperature at which patients consume them. Many recommend that ONS be chilled to fridge temperature before consumption, which has been reported to improve flavour perception (17). Den Uijl et al (17) reported that most older adults in the community consumed ONS preferably at fridge temperature (60 %). However, within hospitals and care homes, there is doubt over the availability of resources for chilling large amounts of products for residents; Den Uijl et al (17) found that patients recieve only 45% of ONS at fridge temperature in care homes. The temperature at which patients drink ONS may impact ONS palatability because ONS served at lower temperatures are less sweet (40). Furthermore, chilled ONS may be more ‘mouth wetting’ (16). Serving temperature may also alter the partitioning of volatile aroma compounds released from the matrix. This may mediate the intensity and quality of the ONS aroma and potentially alter the perceived flavour (41).

### Age and sensory decline

Hubbard et al (14) found a significant negative correlation between adherence to ONS and mean patient age (p = 0.01), suggesting that adherence is worse in older patients. A study by Miller et al (12) support these findings, it was found that participants aged 70–84 years were able to consume 78 % of the prescribed volume compared with 45 % in those aged 85 years and above. Studies have also reported lower adherence in undernourished patients (42, 43). Taken together, it would seem that those most in need of ONS support have the lowest intake (38).

We may expect these findings considering the many factors involved in the “anorexia of ageing”, such as sensory changes, which can impair nutritional intake and could impair adherence to ONS and attempts to improve their nutritional status (2, 44).

For example, olfactory impairments are present in a quarter of older adults, which rises further to 62.5 % in 80–97-year-olds (45). Older adults may also experience changes to their oronasal physiology, such as a reduced salivary flow rate (46) and impaired dentition (47, 48), which may alter their ability to consume and enjoy foods and beverages such as ONS. Age-related sensory impairments may impede appetite, nutritional intake and negatively affect food enjoyment (49, 50, 51, 52).

Research has shown an association between ageing and the perception of ONS; Kennedy et al (53) found that older adults who have higher sweetness thresholds, perceived the sweetness intensity of ONS to be less intensive and rated an ONS more negatively for liking. A non-exhaustive list of age-related changes that may affect adherence, intake and perceived palatability of ONS, deserving of future research, are summarised in Table 1 (adapted from the recent publication of Merchant, Woo and Morley (54))

**Table 1:** Age-related changes that may affect consumer adherence, intake and perceived palatability of ONS

	**Proposed influence on ONS adherence, intake and/or palatability**	**References**
Appetite factors		
Delayed gastric emptying and reduced stomach fundal compliance	• Early satiety and prolonged postprandial satiety	(54, 55)
Dysregulation of appetite hormone release and action	• An inhibition of hunger and early satiety along with delayed gastric emptying	(54, 55)
Reduced physical mobility and reduced energy expenditure	• Reduced appetite	(54)
Altered taste perception and reduced acuity	• Reduced promotion of hunger and enjoyment of eating	(54, 55)
Altered olfactory perception and reduced acuity	• Reduced promotion of hunger and enjoyment of eating	(54, 55)
Oronasal physiological factors		
Altered dentition	• Ill-fitting dentures can create oral discomfort and hinder nutritional intake • Dentures may alter texture and flavour perception	(47, 54, 56, 57)
Other factors		
Frailty and sarcopenic changes and a reduction in strength and physical performance	• Functional difficulty such as reduced ability to lift bottle of ONS and/or remove lid due to lower grip strength and dexterity hindering consumption	(59)
Medication/treatment and polypharmacy	• Exacerbates dry mouth, sensory changes and/or reductions in appetite	(54)
Cognitive impairments	• Confusion and/or difficulty remembering to take ONS in line with prescribed dosage • Declines in mental health are associated with a loss of appetite	(54, 60–62)
Health and disease state	• Reduced appetite caused by infection, medication, or polypharmacy • Neurodegenerative disease and inhibition of appetite	(54)

### Familiarity

Laureati et al (63) suggested that one of the main factors influencing institutionalised older people's food preference may be familiarity and food tradition. These preferences depend on cultural background and previous food experience and vary across individuals and regions. For example, Gosney (11) noted that when British adults were offered a choice of drink, they were more likely to choose tea, a familiar and customary drink in the UK. However, several studies provide somewhat contradictory evidence indicating ONS are well accepted and selected from various high-energy foods and drinks (25, 64).

#### Evidence summary, evidence quality and recommendations

Evidence suggests that the way that patients view and consume ONS may influence palatability and adherence. For example, those living in the community generally prefer it when ONS is framed as a snack food, rather than a medicine, and prefer to drink ONS out of a bottle using a straw. Likely due to greater dependence on healthcare staff, the adherence of more unwell patients (such as those with cognitive impairments) may benefit from ONS being framed as medicine and receiving ONS with medical rounds. Research also suggests a relatively greater proportion of older adults in case homes (compared to the community) prefer to drink ONS from a glass. Nevertheless, likely due to familiarity, strong evidence from a randomised controlled trial found that if ONS are provided in the patients usual drinking method, adherence is higher, highlighting the importance of personalising patient care. Future research should investigate whether communicating personally relevant factors and motives, such as prolongation of independence and strength, or improvements to quality of life, result in tangible improvements in adherence to an ONS prescription. Age-related changes and sensory decline likely further distort the perception of ONS, with many physiological changes impacting palatability and intake. The impact of these changes on ONS intake is deserving of future research and highlights the need for all medical professionals involved in an older person's care (such as nurses, speech and language therapists, medical doctors and carers) to consider older patients' individual needs. Healthcare staff can achieve this through conducting validated appetite screening tools for the ‘anorexia of ageing’, such as the ‘Simplified Nutritional Appetite Questionnaire (SNAQ)‘ (54), along with an assessment of wider factors such as medication use, sensory abilities, cognitive and social health and physical abilities.

In line with the previous section, much of the evidence in this section is observational and in nature, including cross-sectional studies with a relatively low number of participants (n < 100). The feasibility of recruiting a large number of suitable clinical patients for research can be challenging, so these studies do offer a valuable insight of patient experience in a relevant environment and may reflect authentic motives and attitudes.

## Product factors

Product factors can include the type of ONS and product attributes such as thickness, flavour and smell, that largely govern the ONS palatability.

We can define food palatability as the positive hedonic evaluation of foods sensory characteristics, which is strongly determined by sensory properties inherent to the food, and correlates strongly with product intake (65). Therefore, if ONS adherence is to be good, the product properties creating the product's sensory experience must be acceptable to the consumer. Appetite sensations also govern nutritional intake; satiation is defined as the process that leads to termination of eating whilst satiety is defined as feeling of fullness that persists after eating (66). Ideally, the product should have minimal impact on both of these factors to prevent hindering adherence to ONS in the short-term (the consumption period) and subsequently not restrict food and nutrient intake in the longer term (e.g. over the course of a day).

### ONS type

Most ONS are liquid feeds, although powders as well as pudding and bread formats are also available. To meet a wide range of patient preferences and needs, there are various flavours and a range of ONS styles, such as juice, milkshake, yoghurt and savoury ONS (7).

Darmon et al (67) conducted a study to investigate preferences for different varieties of ONS with undernourished in-patients (mean age 64.8 years). It was found that overall pleasantness was significantly better for milk-based than for sweet fruit-juice typed (p < 0.01) and salty juice typed ONS (p < 0.0001).

Taste or flavour fatigue is a frequent complaint of patients, believed to hinder adherence to a full prescription of ONS (16, 68) Taste fatigue is described as a taste driven loss of desire to consume a particular food (69) and occurs due to prolonged and repeated consumption of ONS. One study by Bolton et al (68) found 19% of patients stopped a trial due to reported ‘flavour fatigue’, which accounted for a sizable proportion of those who discontinued the trial early. It is well accepted that in younger individuals, variety can stimulate food intake (16) and through a systematic review, Hubbard et al (14) found that offering a variety of ONS flavours will support adherence, perhaps due to a lower perceived flavour fatigue.

Interestingly, studies varying ONS flavour rather than ONS type showed a greater mean compliance to ONS (14) but healthcare staff should explore both routes to reduce boredom, taste fatigue and potential uplifts in compliance, along with altering of taste of ONS (through different flavours, temperatures and consistencies) (34).

Some authors have commented on the differences in acceptability for supplements prepared freshly with milk against ready to use, long-life supplements with preference given to fresh, milk-based supplements (67, 70, 71). However, it is not clear whether these long-life supplements are the same as those on the market today. In addition, not all freshly prepared supplements are nutritionally complete (defined as providing all nutrients, in appropriate quantities, to be a sole source of nutrition) or prescribable but can be useful when patients' dietary intake is poor (7).

It may be that this preference is due to the generation of unfavourable mouthfeel sensations and aroma compounds generated in the high-temperature processing of dairy proteins that exist in heat-treated ONS product. Regardless, the suitability of using fresh, milk-based supplements in hospitals and care homes may be limited as a consequence of the time and resources required for their preparation and by their storage time (often 12 months for long-life vs only a few days for fresh). In contrast, long-life ONS are commonly nutritionally complete, with good storage time and ready to use involving no preparation time (14).

### Volume and energy density

Consumption of ONS could generate feelings of “fullness” in the patient (17, 30) which may not only impact on complete adherence to an ONS portion (satiation) but also negatively impact on patient's intake of food throughout the day (satiety).

As Nieuwenhuizen et al (16) has indicted, it is well established that the degree of satiation per calorie caused by isolated macronutrients is in the order protein > carbohydrate > fat. As some ONS products are complete forms of nutrition (providing all essential nutrients (macronutrients and essential micronutrients) in appropriate quantities to be a sole source of nutrition) it is challenging for manufacturers to develop ONS without inducing a satiating effect.

Perception of satiety and fullness is linearly associated with postprandial gastric volume (16, 72). Studies have found that, when comparing milk-based drinks with identical nutritional contents, the incorporation of air and water reduced subjective appetite and nutritional intake at a meal served 30 minutes later (73, 74).

Hence, ONS have been developed to offer the required nutritional content in a small volume and condensed form to reduce feelings of fullness, and hence increase subsequent voluntary energy intake (16). Indeed, evidence indicates administering ONS in a smaller volume, but higher energy density effectively increases ONS adherence; a systematic review by Hubbard et al (14) found that mean percentage compliance to ONS with an energy density of ≥ 2 kcal/ml (n = 8 studies) was significantly higher than an ONS with an energy density of 1–1.3 kcal/ml (n = 21 studies) or 1.5 kcal/ml (n = 12 studies) (14).

### Thickness

Thickness is a textural attribute of liquids, which can be defined as the perceived viscosity of a liquid when in the mouth (75).

Den Boer et al (76) found a 33.3 % increase in total volume consumed of a nutritionally matched thin ONS compared to a thick ONS (resulting in higher nutrient intake), without differences in subjective fullness sensations. The authors commented that this finding might have been due to greater and prolonged orosensory stimulation due to slower consumption or consumer expectations about the food's satiating properties (76). The role of perceived thickness on the perceived satiation of high protein drinks has been demonstrated previously (77).

To reduce the thickness and maintain the appropriate nutrient content without increasing the total volume poses a challenge for ONS manufacturers. Compacting ONS into a smaller volume will typically result in a thicker product as the nutrients become concentrated. Product developers must keep in mind that both volume and thickness can increase satiation and potentially decrease ONS intake and balance a small volume while maintaining a thickness designed to minimise satiating impact.

### Flavour

The overall flavour of a food product is a complex combination of three main sensory modalities: olfaction (the perception of aroma compounds), gustation (the perception of tastants) and trigeminal sensations (78); trigeminal sensations combine perception of texture, mouthfeel, temperature and chemesthesis (irritation by chemical stimuli such as capsaicin in chilli).

Flavour is one of the most critical factors determining consumers' acceptance of foods. The perception of flavour is a fundamental survival instinct that allows humans to evaluate foods' nutritional value and safety (79, 80) and plays a central role in the sensory enjoyment of foods in addition to governing appetite and food intake (65).

Patients report poor flavour quality of ONS, including overall flavour type, unbalanced flavour profile, off notes/taints or undesirable aftertastes and sensations, both anecdotally and, in the literature, is frequently linked to low levels of adherence (11, 12, 28, 40, 53, 67, 81, 82). Numerous studies have found that perception of poor flavour is an important factor limiting product liking and adherence to ONS (15, 44, 53, 67, 83, 84).

Several undesirable sensory attributes, such as taints and mouth-effects, stem from nutritional ingredients used in ONS formulations (85, 86). Furthermore, age-related changes in sensory abilities and physiology likely further modulate the perceived palatability and consumer experience of ONS, demonstrating, a complex interaction between factors inherent to the product (undesirable sensory attributes) and factors intrinsic to the consumer (sensory abilities), which influence the overall flavour and palatability of ONS.

For the purpose of this review, we focus on the separate contribution made by trigeminal stimuli, tastants and aroma compounds to the palatability of ONS. However, it is vital to remember that flavour is a complex construct and that interactions will occur between modalities that drive the overall consumer experience (41).

### Trigeminal stimuli

The trigeminal system is responsible for the perception of food texture, mouthfeel sensations and temperature changes and the perception of chemical irritation, such as that from capsaicin in chilli pepper (41). Texture and mouthfeel are important tactile sensations mediated by mechanoreceptors in the oral cavity which play a crucial role in sensation, perception and the safe manipulation of food (87).

#### Mouth drying

Mouth drying is defined as ‘drying sensation in the mouth’ (88) and is a negative driver of liking in ONS products (81).

The perception of mouth drying during ONS consumption is not static but ‘builds up’ over multiple sips during ONS consumption concurrently with self-reported thirst (82, 85). Methven et al (85) compared the perception of a mineralfree and sweet-suppressed (via addition of the sweetness suppressor lactisole) ONS with a standard ONS. They identified mouth drying increased over sequential sips, but there was no difference between mineral-free and standard ONS; however, the sweet-suppressed ONS elicited more intense mouth drying. They concluded minerals were not the primary source of mouth drying and that multimodal interaction between sweetness and drying plays a role, a finding supported by Norton et al (88).

Methven et al (85) hypothesised that the source of mouth drying in ONS products might be milk proteins. Withers et al (89) found that fortification of milk with both casein and whey protein concentrates significantly increased the perception of mouth drying over repeated sips, which confirmed that the source of mouth drying is dairy protein ingredients. Heat-treatment of dairy proteins play a role in the extent of this effect; Bull et al (86) found higher perceptual intensities of mouth drying with an increased heating time of whey protein beverages.

Mucoadhesion, the binding of milk proteins to the oral mucosa, has been proposed to explain the phenomenon of dairy-protein derived mouth drying in the oral cavity (86, 88).

Older adults appear more sensitive to the mouth drying sensation elicited by milk proteins than younger adults (90) possibly due to reduced salivary flow rates, which occurs with age, disease and medication use (46). Lester et al (58) recently detected a greater build-up of mouth drying during multiple sips of ONS for a group of healthy younger adults with a low saliva flow rate, compared to medium and high saliva flow rate groups.

#### Mouthcoating

Mouthcoating, a textural attribute, defined as the residual food that sticks to the oral surface after food ingestion (91) has been studied in ONS products (81, 85). However, mouthcoating is not necessarily a negative attribute. If the sensory qualities of the ONS are well liked, then mouthcoating is desirable (90). Conversely, if the ONS' sensory properties are unpleasant, mouthcoating becomes undesirable. This may be especially relevant for older adults who can experience reduced salivary flow rates and impaired muscle strength for clearing product from the oral cavity (81, 90).

#### Chemesthesis

One recent study investigated the acceptability of ONS prototypes that had the addition of chemical agents that elicit chemosensations, such as cooling menthol and warming/spicy ginger and mango, in patients who were undergoing cancer treatment (92). Patients rated three flavours (cool red fruits, hot mango and hot tropical ginger). Interestingly, one flavour (cool red fruits) was rated significantly higher for liking by patients with taste and smell alterations, compared to patients without taste and smell alterations. Hence, adding chemosensory stimuli to ONS may be an effective way to improve the flavour and palatability of ONS for the older consumer (93), compensating for sensory losses, and warrants further investigation in longer-term clinical studies to evaluate the impact of chemesthesis modification on adherence.

### Tastants

Several studies have provided evidence suggesting the high sweetness of ONS may be a factor limiting palatability and adherence (11, 53, 94)

However, the hypothesis that high sweetness drives a dislike of ONS is not consistently supported by evidence. Methven et al (85) found that an ONS with higher sweetness led to higher mean initial liking compared with an ONS in which the sweetness was suppressed. In addition, compared to an ONS that was not sweetness-suppressed, liking of a sweetness-suppressed ONS decreased more significantly over the consumption of consecutive aliquots (85). Den Boer et al (76) found that participants consumed 8 % more of a sweeter ONS, which also had significantly greater product pleasantness, liking and wanting. Individuals have wide-ranging variation in their optimally preferred concentration of sweetness and researchers can categorise consumers as ‘sweet likers’ or ‘sweet dislikers’ depending on their tolerance for sweetness in foods (95). This may be a factor that could play a part in the discrepancies between findings. Although, much of this experimental research was conducted with healthy individuals, so more research is needed with patients, and ONS-users, to uncover the role of sweetness on ONS acceptance and adherence.

It has been hypothesised that minerals added to ONS during manufacture, such as iron sulfate, may contribute to metallic tastes (85) and are negative drivers of liking in ONS (81). Methven et al (85) investigated the impact of mineral content of ONS and found the mineral free ONS was rated significantly less metallic compared to the control, however the difference was small, and the authors concluded that although the minerals added to the ONS formulation contribute, other components, such as the calcium and milk proteins, may contribute to metallic taste in the products. Researchers should consider investigating changes to commercial products, due to advancing shelf-life, to evaluate whether perceived metallic taste increases in intensity at later shelf-life, potentially due to chemical changes (such as oxidation) of ONS ingredients.

The authors suggested that calcium salts may hold some responsibility because they can exhibit metallic taste properties (85). However, metallic ions can oxidise salivary proteins in the oral cavity resulting in the production of aromatic carbonyl compounds (96, 97, 98). This metallic off-taste is an example of how the addition of essential nutritionally functional ingredients can create sensory challenges in food products (for a review, see Delompre et al (97)).

### Aroma compounds

Aromas are volatile compounds released from foods which stimulate the olfactory receptors in two distinct ways and in doing so play significant roles in food intake. Firstly, orthonasal olfaction (the perception of food aroma before a person places food in the mouth) is critical for evaluating the suitability of food for ingestion (99) and therefore is an essential gatekeeper to food choice and intake. Orthonasal smell drives food acceptance as it occurs prior to consumption, thereby setting our expectations of food palatability (100), modulating appetite (101, 102) and stimulating physiological response (such as salivary flow) in preparedness for food digestion (100). To promote appetite and nutritional intake, positive orthonasal cues are crucial for older individuals that are already experiencing blunted appetite sensations. Positively perceived aromas may be a way to stimulate saliva flow before food consumption in older individuals experiencing hyposalivation. On the contrary, an unpleasant aroma can limit consumers' willingness to consume foods (103). Consequently, ONS should have a palatable, product-congruent and enticing aroma (for example, aroma associated with freshness) to stimulate the patient's appetite and promote the desire to consume ONS.

However, research shows that the aroma of ONS is not optimal. In a series of focus groups conducted with health professionals, Lambert et al (28) identified ‘unpleasant odour’ as a barrier to ONS consumption in patients. In a separate study, through use of a questionnaire, Uí Dhuibhir et al (104) found the sensory attribute ‘smell’ to be one of the least favourite sensory characteristics of ONS as rated by dietitians, and some participants reported a ‘medicinal’ or ‘synthetic’ smell. Healthcare staff who dispense ONS perceive aroma because it is noticeable without consuming ONS. Staff have reported that ONS are ‘not appetising to look at or smell’ (15), which could influence patient expectations before consumption.

The source of unpleasant aromas within ONS has not been fully elucidated. However, it could stem from essential nutritional ingredients within ONS, such as proteins, and/or processing conditions during ONS manufacture. For example, most ONS are heat-treated to ensure consumer safety and prolong the shelf-life but heat-treatment of protein ingredients can cause a greater intensity of ‘cooked’ flavours in highprotein dairy beverages (86). Recent research identified eggy, rancid and sulfate flavours within baked protein-fortified foods (105) and one recent study has identified sulfurous off-flavours within a commercially available ONS (106). Undesirable smelling sulfurous aroma compounds, such as dimethyl sulphide and methanethiol, form in dairy products at high temperatures from essential amino acids present in milk proteins (such as methionine) (107, 108). The generation of unpleasant aroma during heat-treatment could explain the difference in acceptability between ‘fresh’ ONS products and those processed by high-temperature treatment to become longlife ONS.

Retronasal olfaction refers to the release of volatile aromas from foods, which travel through the gaseous airspace, and bind with olfactory receptors in the back of the nose during food consumption. It is retronasal olfaction that combines with the perception of taste and trigeminal stimuli to drive the perception of food flavour, and hence the palatability of food (101).

Researchers associate a greater intensity or duration of stimulation by aroma during food consumption with greater satiation (109, 110). Therefore, an appetising combination and optimal intensity of aroma is crucial for good palatability, adequate intake, and best possible adherence.

Impairments in the older consumer's sensory abilities, which can occur with ageing, disease state, and medication use, likely distort both orthonasal and retronasal perception of aroma. One recent study has found an association between a lower stimulated saliva flow rate, greater in-mouth aroma release and a higher aftertaste perception during the consumption of a commonly prescribed ONS (58), which may have adversely affected appetite.

Many studies have linked age-related declines in olfactory perception to dietary changes and reduced nutritional intake (49, 50, 51, 52, 111). One study found a correlation between reduced smell function in cancer patients with a decreased liking of vanilla-flavoured, milk-based ONS, which the authors attributed in part to a higher smell threshold influencing the palatability (112). In addition, one recent study, Lester et al (106) identified impairments in olfactory abilities in a group of healthy older adults for specific aroma compounds identified as crucial for ONS flavour. No researcher has yet investigated how specifically modifying the aroma contribution within ONS can affect palatability, although aroma enhancement of foods for the older person has produced conflicting results (113).

#### Evidence summary, evidence quality and recommendations

Undernourished older adults have shown preference for milk-based ONS over juice-based and there is limited evidence that consumers prefer freshly-prepared ONS over long-life ONS. The latter however are more convenient to dispense, requiring little to no preparation time and more convenient in clinical settings. Strong evidence shows that a smaller volume ONS with greater energy density promotes greater nutritional intake. However, manufacturers must consider that thickness can also induce satiation. So, manufacturers of ONS must consider both factors and identification of the optimal balance between thickness and volume is an evidence gap demanding of future research. Providing a variety of flavours and ONS types may further support patient adherence by reducing taste fatigue and boredom. Finally, manufacturers should strive for optimal sensory characteristics, including minimising undesirable off-flavours and mouthfeel taints, but also by incorporating enticing, appetite-stimulating aromas and flavours.

In particular, we know little about the contribution made by aroma compounds to the flavour and palatability of ONS or how age-related changes in physiology and sensory abilities of older adults further distort the perception of, and adherence to, ONS. Due to the importance of aroma in food acceptance, palatability and intake, this gap in the evidence requires further investigation through well designed and randomised controlled trials. Methodology employed should comprise validated sensory standards (such as those standardised by the American Society for Testing and Materials (ASTM)). Studies should be appropriately powered and could consider including both healthier older adults and/or undernourished older adults as study subjects. Albeit, undernourished older adults are the end-consumers of ONS and understanding this group's product experience and preferences is ultimately key for ensuring adequate nutritional intake in this group.

As shown in the previous section, evidence to show that older adults experience physiological and sensorial changes that affect nutritional intake is strong, however due to only a small amount of research existing, we can currently only make a limited number of strong recommendations to show how manufacturers can optimally design ONS to meet older patient needs. Strong evidence does support offering ONS in a small volume with high energy density in addition to prescribing patients a variety of ONS (particularly a variety of flavours). Strong evidence also supports the personalisation of patient care, including in offering a choice of ONS to meet preferences, needs and familiarity. Further research should aim to affirm findings discussed, through well-controlled randomised trials, to establish firm guidelines for ONS provision and for the optimal design of ONS formulations.

## Conclusion

With the identification of key factors influencing adherence and strategic expansion of research effort, there are exciting opportunities to develop the next-generation ONS products. Three distinct domains relating to adherence were identified and, on the basis of this, we have made recommendations to optimise each domain.

Firstly, contextual factors were identified. This review highlighted the importance of educating and unifying health care professionals and educating family members with respect to the benefits of ONS, encouraging positive communication of these benefits and providing greater confidence in the consequences of their use; this could potentially promote patient adherence to ONS.

Secondly, recognising personal preferences in product presentation, as well as individual physiological capabilities, and provision of options for consumption (glass, straw, etc) may also be beneficial.

Thirdly, with respect to the product domain, increased palatability, through modification of texture and use of flavour profiles explicitly designed for older sensory abilities, will contribute to increased end-consumer adherence. Careful balancing of viscosity, thickness and volume of products would deliver multiple advantages; reducing satiation and satiety (and therefore impact on subsequent food intake), whilst providing nutritional benefits in a smaller volume designed to minimise gastric bloating. Advances in understanding the mechanisms behind mouth drying sensations may provide additional opportunities to reduce this key driver of dislike in ONS products, whereas chemesthetic stimuli could offer routes to deliver oral sensations which enhance appeal. One comparatively under-researched area is the contribution made by aroma to older adults' experience of ONS and further research should address this gap. Ortho-nasal aroma perception drives initial expectation and manufacturers should exploit this to maximise desire to consume. In addition, learnings from aroma-induced taste, texture and appetite modification studies should be leveraged so collectively enabling optimised flavour profiles, which stimulate appetite and salivation.

Combined, these approaches will ultimately elevate the palatability of ONS, increase consumer intake and adherence to prescribed therapeutic interventions. Thus, we can maximise the clinical benefit of nutritional supplementation in the ageing population.
